# Septicæmia Hæmorrhagica

**Published:** 1896-10

**Authors:** 


### ABSTRACTS FROM FOREIGN JOURNALS.

UNDER THE DIRECTION OF

J. PRESTON HOSKINS, PRINCETON, N. J.

SEPTICÆMIA HÆMORRHAGICA. In the middle of September Government Veterinarian D. Hubenet received a telegram that a disease had broken out among the buffaloes in the department of Soemedang and in the district Dermawangi (Dutch East Indies). He proceeded to the place where the disease was most prevalent, and learned the following facts :

The first case of the disease had showed itself on September 20th in a buffalo out of a large herd which was grazing on the plain to the east of the ranch Marioek, whither it had been brought in order to water it in the river Tjipelung. The buffalo mentioned, without showing any previous signs of sickness, fell down and died suddenly. The head of the district, known as a shrewd and zealous official, had the dead animal buried as soon as he learned of the occurrence, and brought the rest of the herd under cover near the ranch, where the animals were carefully provided for and the grass thoroughly cleansed before it was given to them. For a long time the district had not been visited by rain, so that everything was burned and dry; the little grass which the sawahs (or meadows) produced was covered with a layer of dust. Shortly after isolation the disease broke out among the buffaloes in the kraal; animals which up to that time had appeared healthy now fell down and died. In the meantime all other animals which did not belong to the infected herd were, as a matter of precaution, removed to a safe distance. Through these measures the disease was confined to the herd first attacked, from which twenty-one animals died before September 15th. At the time of Hubenet's investigation the next day he found that at another village, Palasah, a buffalo had died, while two sick animals were still standing in the kraal; at Marioek the number of dead had reached twenty-eight, and two more animals which showed signs of sickness about three hours before died in his presence. Besides the dead animals eight others stood in the kraal which were more or less sick, or at least suspected of being infected. It was plain from this that certain abnormal symptoms had been noticed by the owners which were constantly present in the animals which later fell sick or died suddenly. The native owners of buffaloes know their animals exceedingly well, especially as they do not possess, as a rule, more than three or four head. They had noticed lately little knobs (granulations) or swellings on their cattle which they had never seen before. These granulations increased in size, sometimes being not larger than a beetle-nut and then becoming large, doughy swellings which either appeared on the whole of one of the fore or hind legs and covered the throat, or spread itself along the belly, breast, and neck. Becoming aware that such appearances were the beginning of a course of disease, they isolated the beasts.

Hubenet had ample opportunity to observe the disease closely at Marioek, where there were eight sick and two dead buffaloes. It was evident that swellings of the skin of different sizes were present in all the sick animals. The smallest of these swellings had the appearance of little knobs or pimples; and the larger they were the sicker the animal, so that while some animals still continued to eat heartily others suffered severely. In regard to the last, it must be noted that evacuations had entirely ceased as well as rumination; no trace of movement in the paunch was noticeable. The temperature in some animals rose from 40° to 42°; defecation scanty; the dung was hard and of a dark color; only in one case did it assume a thin and bloody consistency. In some animals the mouth was half open, and the tongue, which was very much swollen, protruded; an excessive secretion of saliva was present in some cases, causing a snoring sound due to the swelling of the mucous membrane of the larynx, over which, as a section showed, there was a thick, tough slime. Besides all this the animals leaned over against objects continually for support, often lay down, and where they continued to stand seemed to be on a continual strain as if a spell of colic was attacking them at the same time; very speedily, however, inability to hold up overcame them, and after they sank down death quickly followed. It is certain that the course of the disease would have been noted earlier if one had attached importance to the little swellings whose significance had in general been learned earlier. But nothing else was noted than the sudden falling down and death, and, strangely enough, each succeeding day buffaloes could be seen dying which had passed through a long course of disease; even cases of recovery occurred in animals which were seriously afflicted. Could this be due to the fact that the herds brought into the kraal at Marioek lived under more favorable circumstances, as far as food and water were concerned, than those out on the plain, and that in consequence the organism causing the disease did not enter into the body in such great numbers? or have we to do with the phenomena that an infectious disease at its beginning pursues a more acute course than when it has been in existence for a long time? In Hubenet's opinion the principal factor in this was the better care which the animals received.

On making a post-mortem he found the skin of the stomach swollen in places and the subcutaneous tissue infiltrated with a serous exudation; the whole had the appearance of a jelly-like mass. A thin bloody fluid flowed off from the cavity of the stomach in great quantities; the peritoneum showed here and there a well-defined severe infection with loose adhesion of parts of the surface of the paunch. Everywhere here and in the mesentery were present flat hemorrhages, varying in size from a silver dollar to that of the palm of the hand. The spleen and liver were normal, likewise the paunch, but the whole mucous membrane of the stomach was infected to a high degree. The kidneys were of normal size, with a little bleeding in the tissue just beneath the capsule. The mucous membrane of the small intestine had the appearance of a hemorrhagic enteritis, while the lumen was partly filled with coagulated blood; Peyer's glands were somewhat swollen, but the mucous membrane of the rectum was normal. That of the urino-genital system showed small spots here and there. In the thoracic cavity he found the pleura normal; here and there hemorrhages were present in the interstitial tissue of the lungs, in the bronchial and tracheal mucous membrane, and in the mediastinum, besides in the muscles of the heart and in the inner surface of the peri- and endocardium. In the bronchiæ and trachea, and further in the larynx, a thick, tough slime was present, which filled the glottis up considerably and often gave rise to difficulty in breathing. The lung tissues were normal, only containing blood here and there in the interstitial tissue. The nostrils were likewise partly filled with the same thick slime which from time to time was ejected in thick masses. His diagnosis was septicæmia hæmorrhagica, more definitely the exanthematic form, in many cases complicated with the intestinal form. Many of the cases were subacute or acute, and he did not always have an opportunity to make a post-mortem examination. However, he did make examinations of animals that had suddenly died and found the same phenomena that had been described above. In most cases the tongue was swollen and hung out of the mouth, so that he first thought of anthrax, but the microscopical examination always gave a negative result. The epidemic often occurs in Java, and especially in West Java, and the cattle trade has suffered greatly from it. At times the epidemic occurs among the wild animals in Soemedang, as well as among the deer and hogs, and causes great destruction, as may be seen from the many cadavers which are found in the woods. The natives have already connected the disease with that among the buffaloes.

As to the methods of fighting the disease, the first was to quarantine the sick and suspected from the healthy ones. This quarantine was carried out with great strictness. This measure was partially justifiable, as there was but little and very poor grass on the pastures. The grass fed to the animals could then be carefully washed. Carbolic acid was used as a means of disinfection, but the application was made in such a way that little effect could be expected from it. On the whole, a considerable number of cases recovered. In Marioek at least ten out of the fifty cases got well; these all belonged to the herd in which quarantine had been exercised as soon as the first case had been noticed. The sudden disappearance of the disease as soon as quarantine measures had been adopted and good water and good grass had been given to the animals seems to show that mutual infection from one animal to another is not to be feared either directly or indirectly, just as is the case in rinderpest and in the foot-and-mouth disease; but the cause is to be looked for in some infectious matter, the conditions for which are to be found alone in the soil. Although he sent preparations to the pathological-bacteriological laboratory at Weltoreden, no result could be obtained as the great distance had brought about changes in the condition of the preparations which rendered them worthless.—*Veeartsenijkundige Bladen voor nederlangsch-indie*, Deel ix. Aflevering 4.

